# Supplementary material for: From sequence to enzyme mechanism using multi-label machine learning
Source: BMC Bioinformatics. 2014 May 19;15:150. doi: 10.1186/1471-2105-15-150 (PMC4229970; doi:10.1186/1471-2105-15-150)
Supplement: Additional file 2 — Java code of ml2db. Additional file ml2db_code.tar.gz contains the Java source code to run the multi-label machine learning experiments and save the results to database. The code’s Javadoc is included. [file 1471-2105-15-150-S2.zip › additional file 2/ml2db/ml2db/doc/mulan/transformations/multiclass/class-use/MultiClassTransformationBase.html]

Uses of Class mulan.transformations.multiclass.MultiClassTransformationBase


JavaScript is disabled on your browser.


- Overview
- Package
- Class
- Use
- Tree
- Deprecated
- Index
- Help

- Prev
- Next

- Frames
- No Frames

- All Classes

## Uses of Class mulan.transformations.multiclass.MultiClassTransformationBase

- Packages that use MultiClassTransformationBase

  | Package | Description |
  | --- | --- |
  |  |  |
  | --- | --- |
  | mulan.transformations.multiclass |  |
- - ### Uses of MultiClassTransformationBase in mulan.transformations.multiclass

    Subclasses of MultiClassTransformationBase in mulan.transformations.multiclass

    | Modifier and Type | Class and Description |
    | --- | --- |
    |  |  |
    | --- | --- |
    | `class` | `Copy` Class that implement the Copy transformation method |
    | `class` | `CopyWeight` Class that implement the Copy-Weight transformation method |
    | `class` | `Ignore` Class that implement the Ignore transformation method |
    | `class` | `SelectBasedOnFrequency` Class that implement the Select-Max and Select-Min transformation methods. |
    | `class` | `SelectRandom` Class that implement the Select-Random transformation method |

- Overview
- Package
- Class
- Use
- Tree
- Deprecated
- Index
- Help

- Prev
- Next

- Frames
- No Frames

- All Classes
